# Recent Increased Loading of Carbonaceous Pollution from Biomass Burning in the Baltic Sea

**DOI:** 10.1021/acsomega.2c04009

**Published:** 2022-09-23

**Authors:** Karl Ljung, Petra L. Schoon, Marcus Rudolf, Laurie M. Charrieau, Sha Ni, Helena L. Filipsson

**Affiliations:** Department of Geology, Lund University, Sölvegatan 12, Lund 223 62, Sweden

## Abstract

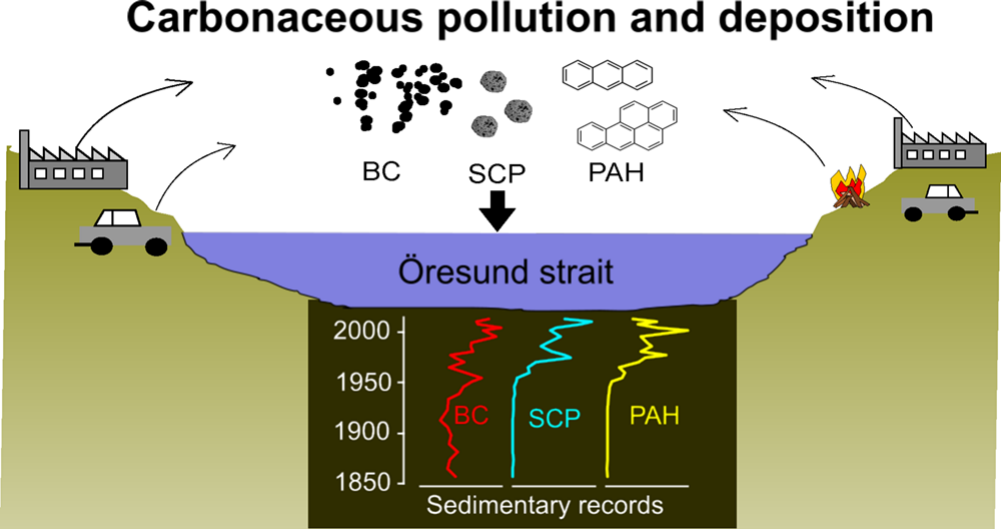

Black carbon (BC), spheroidal carbonaceous particles (SCP), and polycyclic aromatic hydrocarbons (PAH) are carbonaceous pollutants affecting the climate, environment, and human health. International regulations limit their emissions, and the present emissions are followed by monitoring programs. However, the monitoring programs have limited spatio-temporal coverage and only span the last decades. We can extend the knowledge of historical emission rates by measuring pollution levels in radiometrically dated marine and lacustrine sediment sequences. Here we present measurements of BC, SCP, and PAH from a sediment sequence sampled in the Öresund strait, between Denmark and Sweden and dated back to CE 1850. Our data show a massive increase in the burial rates of all measured pollutants starting in the 1940s. The pollution deposition peaked in the 1970–1980s and declined through the 1990s. However, the declining trend was reversed in the 2000s. Source appointment of PAHs shows a relatively higher contribution of emissions from wood-burning since CE 2000. This coincides with a change towards the increased use of biomass for both municipal and regional energy production in Scandinavia. Our results demonstrate that changes in energy production have caused changes in the delivery of carbonaceous pollution to marine environments. The increase in particle emissions from wood burning is potentially posing a future environmental and health risk.

## Introduction

The burning of fossil fuels and biomass emits polycyclic aromatic hydrocarbons (PAHs), spheroidal carbonaceous particles (SCPs), and black carbon (BC) into the environment. PAHs and BC are produced both naturally, e.g., in forest fires, and anthropogenically by the combustion of fossil fuels, while SCPs are only produced by the high temperature combustion of fossil fuels. The emissions have severe health consequences with over four million deaths per year estimated to be caused by air pollution globally,^1^ and particles from biomass burning being of particular concern.^2,3^ Carbonaceous particle emissions also affect the climate and BC is a major greenhouse substance.^4−6^ The emissions of carbonaceous particles and compounds are highly variable over both time and space, and airborne particles can travel across geographical borders and reach areas far from their sources.^7−9^ The current emission patterns are in many cases well characterized by monitoring programs.^10,11^ The long-term changes in emissions are, however, not well captured in monitoring programs as they typically only cover the last decades. The long-term trends of carbonaceous particle and PAH emissions are important for understanding their history, tracing the fate of pollutants in the environment, and determining the effects of environmental regulations on emission patterns.

During the past decades, there has been a general decline in particulate emissions from many sources such as road traffic and industries due to stricter environmental regulations.^12,13^ However, concerns are currently rising that emissions from biomass burning are increasing, and it has been shown that emissions of carbonaceous particles from domestic wood burning are significant and a growing health concern in Europe.^14,15^ In Scandinavia, studies from Denmark and Sweden have demonstrated that emissions from residential wood burning contribute significantly to particle emissions with potential adverse health effects.^16,17^ Increased particle loadings from wood burning in Sweden in the 1990s and 2000s have also been inferred from reconstructions based on PAH and BC concentrations of lake sediment archives.^18^ Reconstruction and understanding of the long-term trends on decadal to centennial or even millennial time scales are crucial for a systematic understanding of the processes determining the environmental load of organic pollutants.

**Figure 1:**
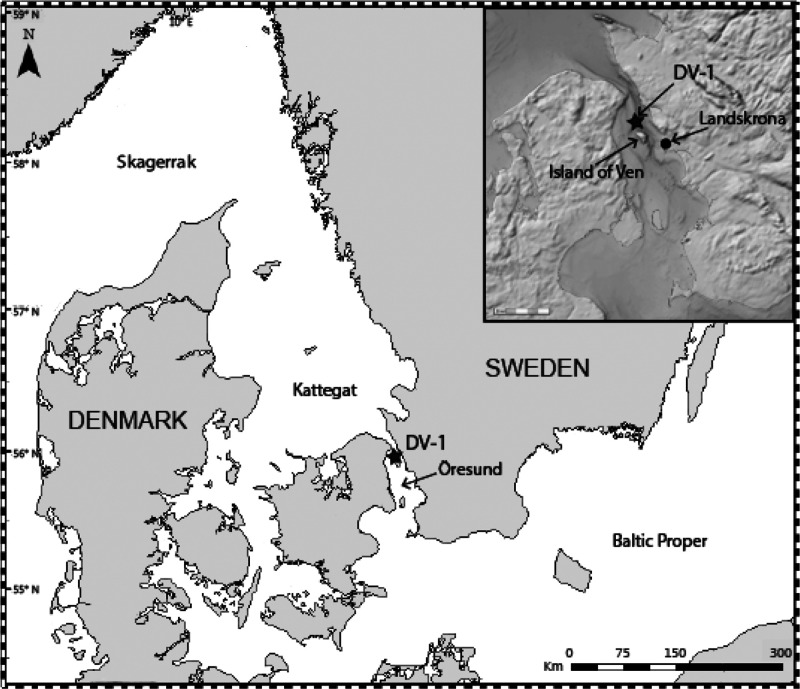
Map showing the location of the coring station DV-1. Adapted with permission from (Charrieau et al.)^20^ under creative commons license: http://creativecommons.org/licenses/by/4.0/.

Here we present sedimentary concentrations and deposition rates of PAH, SCP, and BC in the entrance of the Baltic Sea from CE 1850 until 2013 (Figure 1). The surrounding provinces in Sweden and Denmark are densely populated with a population of about four million people. Furthermore, the strait is a major shipping route for vessels entering and leaving the Baltic Sea, with over 30,000 passages of ships annually (ships over 300 grt, not including local (HH) ferries).

We used diagnostic PAH indices to distinguish sources of the carbonaceous pollution and the BC and SCP deposition rates to estimate the deposition of soot and particulate matter. These records provide a unique window into the pollution history of the Öresund region and give a long-term perspective on the modern accumulation of carbonaceous particles and compounds.

## Materials and Methods

### Study Area and Sampling

Öresund is a narrow strait, 4–28 km wide, between Sweden and Denmark, connecting the Baltic Sea with the Kattegat, Skagerrak, and the North Sea (Figure 1). The average water depth in the strait is 25 m and the deepest part is 53 m. The strait is one of three pathways for water exchange between the Baltic Sea and the North Sea, with generally high current velocities.^19^ The high current velocities make finding sites with consistent sediment accumulation challenging in the strait.

Sediment cores were retrieved using the twin-barrel Gemax-corer (9 cm diameter) deployed from R/V *Skagerak* in November 2013 at the Öresund station DV-1 (55°55.59′N, 12°42.66′E) at a water depth of 45 m (Figure 1). The station DV-1 is situated in an area with an accumulation bottom just north of the Island of Ven.^20^ The cores were sliced in one centimeter subsamples with a known volume on-board the ship. The subsamples were immediately frozen after sampling and kept frozen until freeze-drying. The water content and dry density were measured by weighing before and after drying. The grain-size distribution was measured on each subsample (Supplementary Figure S2).^20^

### Sediment Dating

The chronology of the core is based on ^210^Pb and ^137^Cs measurements on samples from a parallel core (DV1-G) that was correlated with the analyzed core using the distinct total organic carbon (TOC) pattern present in all investigate cores. The chronology is previously published in Charrieau et al.,^20^ and details of the dating and chronology are described in the Supplementary Material.

### Soot BC

BC was analyzed using the thermal-chemical-oxidation method (CTO375).^21−23^ CTO375 quantifies the most condensed and resistant form of soot BC produced in high temperature combustion both from fossil fuels and natural occurring fires.^21^ Briefly, the freeze-dried and homogenized sediment was oxidized in a furnace at 375 °C with forced air-flow followed by acid fumigation using hydrochloric acid (Supporting Information). The remaining carbon was quantified using an elemental analyzer (Costech ECS4010). The detection limit was estimated to be 2 μg C based on the response of replicated blank runs. The BC quantification was evaluated against reference materials with the published BC content and ranges were within the ranges of published values (Supporting Information Table S1). The method relative precision was estimated to 15% based on the replicated (*n* = 3) reference material (NIST1944).

### SCPs

SCPs are exclusively produced by fossil fuels in combustion engines and are a part of the BC continuum.^12^ SCPs were quantified following (Rose) ref (24). Samples were treated with HCl, HF, and hydrogen perchlorate to oxidize inorganic and organic materials. The treated residue was transferred quantitatively to a microscope slide. The counting of SCPs was done under a light microscope at 400× magnification. The identification of SCPs was performed using published descriptions^24^ and corroborated by scanning electron microscopy images. Procedural blanks yielded no detectable SCPs. The whole sample was counted, and the counts were back-calculated for the whole sample weight.

### Polyaromatic Hydrocarbons

Freeze-dried sediment samples were Soxhlet extracted with a 7.5:1, v/v mixture of dichloromethane and methanol (MeOH). The total lipid extracts were further separated into apolar, aromatic, and polar fractions using silica gel chromatography (Supporting Information). The PAHs in the aromatic fraction were identified and quantified by gas chromatography (Agilent 7890B) and gas chromatography–mass spectrometry (Shimadzu QP2010 GC/MS). Procedural blanks yielded no detectable target compounds, and the recovery rates were close to 100%. Detailed extraction and chromatographic and mass spectrometer conditions are described in the Supporting Information.

## Results and Discussion

### Historical Fluxes of BC, SCP, and PAH

**Figure 2:**
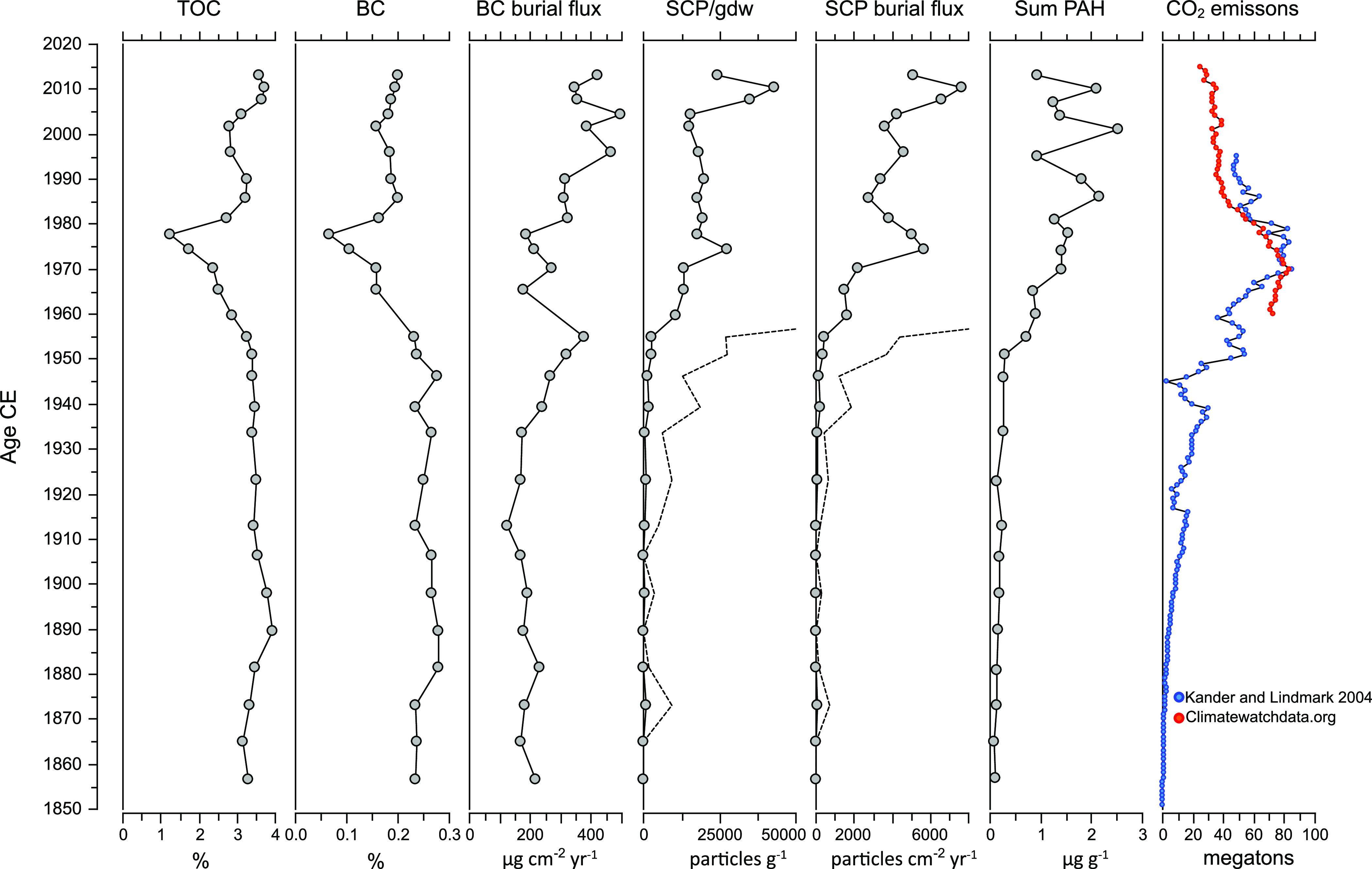
Concentrations and burial fluxes of TOC, BC, SCPs, and sum of all PAHs from the DV-1 core. Dashed lines in SCP burial flux and SCP particles/g show 10× exaggeration of the measured values. CO_2_ emissions from Sweden are derived from Kander and Lindmark^44^ and Climatewatchdata.org.^25^

**Figure 3:**
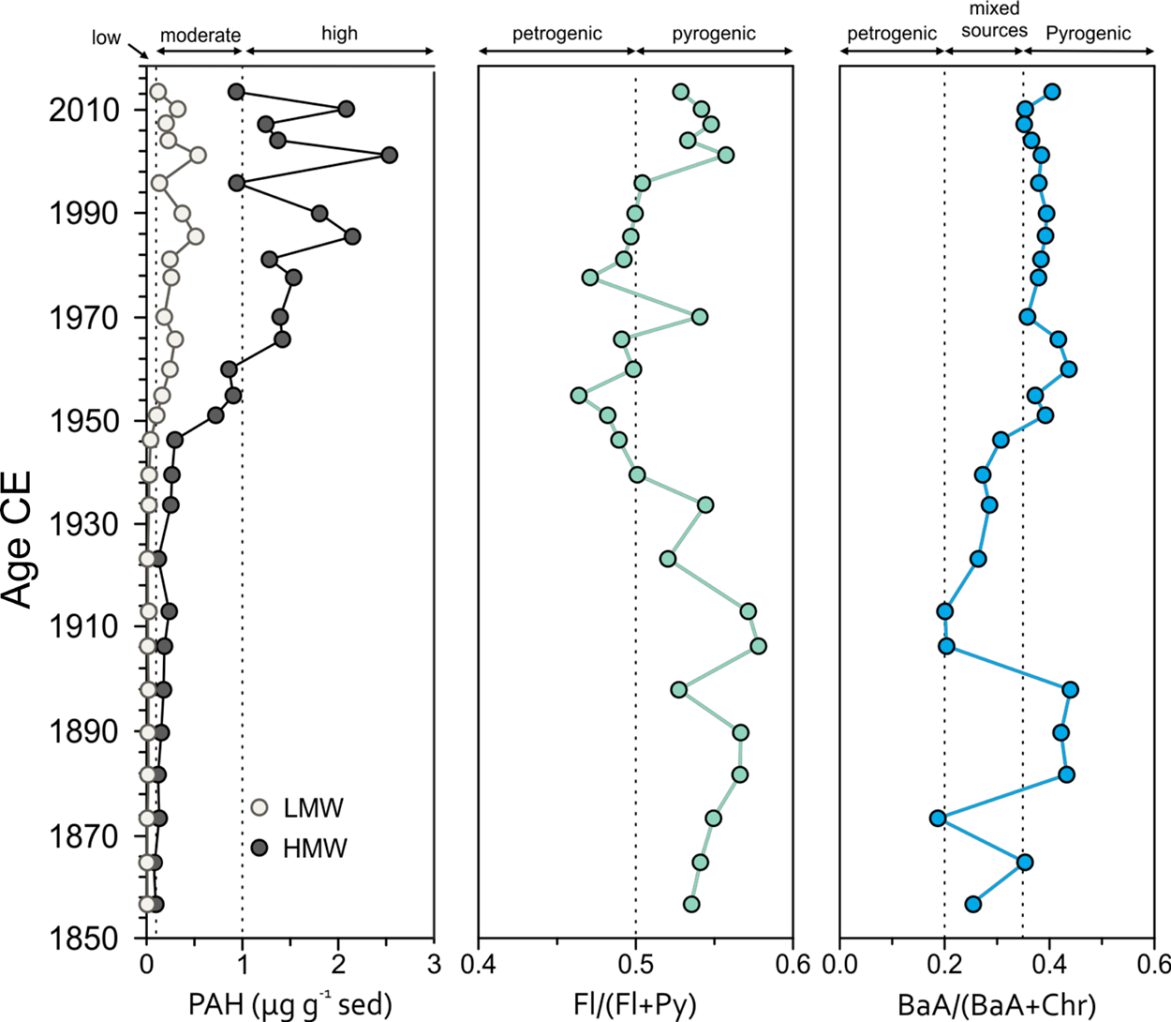
Sum of all polyaromatic hydrocarbon (PAH) concentrations, separated by low molecular weight (3–4 rings) and high molecular weight (5–6 rings), and ratios indicative of pyrogenic (from combustion) or petrogenic (from fossil fuels) sources of the PAHs.

SCPs were not observed in the two lowermost samples, and BC and total PAH concentrations were below 0.25% and 0.1 μg/g (Figure 2). The absence of SCPs and low PAH concentrations indicate that the deposition of pollution from the combustion of fossil fuels was low and that these values represent close to unpolluted background levels. Around 1875 burial fluxes of BC and PAHs increased slightly and we observed SCPs in the sediments for the first time (Figures 2 and 3). BC and SCP burial fluxes increased around 1930 followed by an increase in total PAH concentrations shortly after. From ∼1950, the burial fluxes of SCP and PAH concentrations increased. BC fluxes also increased around this time, but with a reversed trend between 1960 and 1980. Around 1985 SCP burial fluxes reached a maximum followed by a maximum in total PAH concentration centered around 1990. Between 1990 and 2013 BC, SCP and total PAH burial fluxes increased and reached maximum values.

The mean BC concentration in the uppermost two cm of the sediment sequence was 1.97 mg g^–1^, similar to the reported core top values from Öresund.^26^ The mean total concentration of PAH in the top two centimeters was 1.5 μg g^–1^ which is comparable with previously reported surface sediment PAH values from Öresund ranging between 0.06 and 1.65 μg g^–1^.^26,27^

The correlation between PAH and SCP concentrations was high (*R*^2^ = 0.6, *p* < 0.01). SCPs are produced exclusively by the high temperature combustion of fossil fuels (coal and oil) while PAHs are produced both by fossil fuel and biomass burning. The positive correlation between SCPs and PAHs indicates that their sources were similar, and that a large increase in the PAH concentration observed in our record after 1955 to a large degree was derived from fossil fuel combustion. The PAH, SCP, and BC concentrations were negatively correlated (*R*^2^ = −0.5, *p* < 0.01, respectively), which is contrary to the expected pattern, as previous studies have shown a positive correlation between BC and PAH in marine sediments.^26^ Both BC and PAH deposition in marine sediments is linked to the clay content.^28,29^ The negative correlation observed here could be an effect of a coarser material and changing depositional environment from 1960 to 1980 caused by higher flow velocities through the Öresund strait (Supplementary Figure S3).^20^ The coarser material explains the lower BC concentrations, while PAH concentrations did not decline to the same extent. This pattern is different from studies of surface sediments in the Baltic Sea region, where a strong positive correlation has been observed between BC and PAH.^26^ These conflicting patterns indicate that BC and PAH deposition is controlled by different mechanisms spatially and temporally. The spatial distribution is mostly determined by source and transport differences, while the temporal difference observed in our study is also affected by sedimentological conditions with different effects on BC and PAH accumulation. The clay content and BC concentration are positively correlated (*R*^2^ = 0.57, *p* < 0.01), which shows that BC is associated with finer fractions (clay), rather than coarser fractions (>silt) (Supplementary Figure S2). The sorption of BC to fine-grained minerals has been pointed out as being important for transportation in aquatic systems and it has been shown that the BC concentration in lake sediments is higher in the fine (clay) fraction.^30−32^ Thus, it appears that PAH deposition was not affected to the same extent by the higher flow velocities and coarser grain size as BC deposition at this site.

Changes in the sources of the PAHs could also be partly responsible for a weaker correlation between particulate pollution and PAHs. If a larger fraction of the PAHs was associated with char, that is not detected by the SCP counts or by the CTO375 method used for isolating BC, this could explain a weaker correlation. If the PAH source was dominated by direct fossil fuel pollution to a larger extent, the correlation with BC and particulate matter would also be weaker.

The general increases in burial fluxes of BC and SCP, and concentrations of PAHs in the sediment sequence follow the well-established trend caused by industrialization in the 19th and 20th centuries.^12,18,33,34^ SCP are exclusively produced by fossil fuel burning,^24^ and the early increase in SCP concentration around 1870 coincides with the early phase of industrialization in the region and the transition from sail to coal-powered steamships.^35^ The increase agrees well with previously observed increases in SCP deposition in Scandinavia and Europe.^12,36,37^ The increase in BC and SCP burial fluxes and PAH concentrations after 1950 is most probably the consequence of the “Great Acceleration” after the second World War. The rapid economic and technological development led to the rapidly increasing use of coal and petroleum with high emissions of soot and PAH. High concentrations of sedimentary BC, SCP, and PAH have been reported for this time period from other sites in Sweden^22^ and globally.^12^

The traces of the high emissions are clearly visible in our data, with high fluxes of SCP and high concentrations of PAHs between 1975 and 1990 (Figures 2 and 3). After 1990, the fluxes and concentrations level off, but with some variability. SCP burial fluxes declined around 1985, while PAH concentrations declined after 1990 and the BC burial fluxes leveled off in the 1990s (Figures 2 and 3). Many records of BC and SCP show similar trends of declining deposition after 1990 and the general pattern is that the deposition of SCP in most parts of the world continued to decline through the 2000s, excluding more recently industrialized countries.^12,33,38^ The decline in SCP and PAH emissions is usually attributed to the direct effects of improved cleaning of vehicle exhaust and industrial emissions, along with stricter environmental regulations, that have led to a general decline of particulate emissions from anthropogenic sources in Europe during the recent decades.^39^

However, from around 1995, our data indicate a reversal of the decline in BC, SCP, and PAH deposition. Between 2000 and 2013, the highest concentrations and burial fluxes of BC and SCPs were observed in the sediment sequence (Figures 2 and 3). It is intriguing that we observe the highest burial fluxes of SCPs in the most recent part of the sediment core. This might indicate strong local deposition, potentially caused by high local emissions or by more effective transport and burial from distant sources. The intense ship traffic through Öresund could be a strong local emission source and partly responsible for the increased deposition of SCP and soot BC. Ship traffic in the Baltic Sea is known to emit high amounts of particles,^40,41^ and the high SCP fluxes in recent decades could be tentatively linked to the increasing load of particles from marine traffic through Öresund. It is also possible that increased road traffic across the Öresund bridge connecting Sweden and Denmark, which opened in 1999, has contributed to the deposition of particles.

The here documented high burial fluxes and concentrations of BC, SCP, and PAH in the recent decade indicate that the environmental loading of carbonaceous pollution has increased. A similar reversal of the declining trend of organic pollution was observed at a site in northern Sweden, where concentrations of BC and PAHs started to level off in the early 2000s.^18^ Thus, our data indicate that the trend observed in the Öresund may be part of a regional long-term pattern of slightly increasing particle loadings, which might be related to a change in emission sources.

### Sources of Organic Pollutants

**Figure 4:**
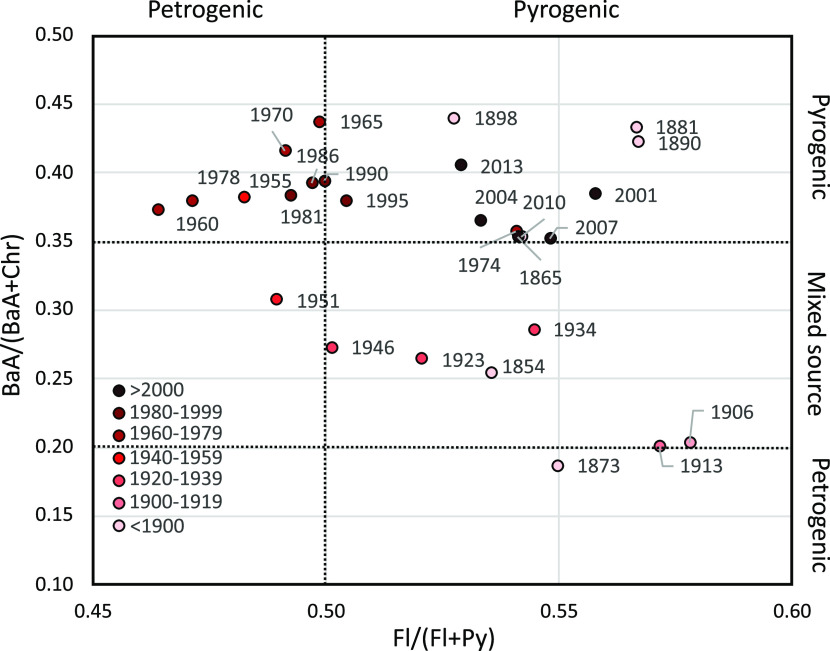
Indices indicative of petrogenic (from petroleum) or pyrogenic (combusted biomass or coal, oil) sources of PAHs. Sample labels and color coding indicate the age of the samples.

We use the distribution of PAHs to distinguish different pollution sources. The ratios of fluoranthene to pyrene (Fla/(Fla + Pyr)) and benzo(*a*)anthracene to chrysene (BaA/(BaA + Chry) are employed to distinguish between petrogenic (from petroleum) or pyrogenic (from combustion of wood, coal, or oil) sources.^42,43^ The PAH indices show three main groupings of the data (Figures 3 and 4). Before 1945, the concentrations of PAHs were low and the ratios must be interpreted with caution, but most samples indicate a mixed source of petroleum and combustion of wood, coal, or oil. After 1945, PAH concentrations increased (Figure 3) and the Fla/(Fla + Pyr) decreased below 0.5 indicating a higher proportion of petrogenic sources and cluster in the upper left corner of the cross plot (Figure 4). The low ratios were interrupted by an increase in the Fla/(Fla/Pyr) during the period of lower clay content between 1960 and 1980. The higher Fla/(Fla/Pyr) ratio indicates less fossil fuel derived PAHs, and this could be a direct effect of sorting of the individual PAH compounds through higher current velocities and coarser materials.

After 1990, the Fla/(Fla + Pyr) increased to high values because of relatively higher concentrations of fluoranthene, and the most of the samples cluster in the top right corner of the cross plot (Figure 4). This shift indicates a higher contribution from combusted coal or wood relative to coal and oil. The shift after 1990 also coincides with higher concentrations and burial fluxes of BC and SCPs (Figures 2 and 3).

The higher PAH concentration in the sediments and indication of combusted liquid fossil fuels being the major source from 1945 can be explained by the increasing use of petroleum products for energy use, and the general industrialization of the region. The increase in pollution from the combustion of fossil fuels continued until the late 1980s. The high PAH concentrations during the 1970s and 1980s coincide with high fossil fuel consumption (Figure 2), as the period coincides with a peak in oil consumption in Sweden.^44^

The higher concentration of PAHs, burial fluxes of SCP and BC after 2000 also coincided with a shift to higher Fla/(Fla + Pyr), indicating a higher contribution from coal and wood combustion. PAHs with 5 and 6 rings also increased and reached the highest concentrations after 2000 (Figure 3 and Supporting Information Table S2). Heavier PAHs (>4 rings) are less volatile and are to a larger extent deposited as a part of the particulate material. The high concentrations of PAHs, SCP, and BC together with the higher Fla/(Fla + Pyr) indicate a higher deposition of soot from wood and biomass burning. Similar changes indicating the increased loading of pollution from wood and biomass burning are shown from sedimentary archives from northern Sweden.^18,22^ Our data show that the trend previously indicated in the early 2000s by Elmquist et al.^18^ from northern Sweden has continued over time and is also apparent in southern Sweden.

The increased loading of organic pollutants and particles occurs at a time when most pollution deposition from fossil fuel burning has declined.^39^ Most PAH and BC deposition was thus expected to also have declined. The evidence for increased wood and biomass burning from sedimentary archives indicates a change in the sources. The likely cause of the increase in the deposition of particles and organic pollutants is an increase in biomass burning for heating. District heating systems in Sweden have changed from relying almost entirely on fossil fuels in the 1970s to predominantly using biomass today, with a steady increase in the number of domestic wood-burning (pellets) stoves for heating.^45^ Copenhagen (Denmark) has high concentrations of particulate emissions coming from wood burning compared to Oslo (Norway) and Umeå (northern Sweden), which is explained by both high local sources, residential wood-burning stoves, and higher background levels.^46^ In Europe, wood burning, mainly coming from heating sources, is the largest source of organic aerosols.^47^ Atmospheric particle monitoring of background levels across Europe also shows that the contribution from wood burning can contribute up to 50% of atmospheric PAH in Sweden.^48^ The energy production from biofuels tripled in Sweden between 1988 and 2018, to a total production of 141 TWh in 2018.^49^ Of the total biofuel consumption in 2018, wood fuel contributed 60 TWh.^46^ District heating is one of the biggest biofuel consumers (37.6 TWh in 2018) and the use has increased steadily from >1 TWh in the 1970s. The shift in our data to higher burial fluxes of BC and SCPs together with a change to a higher proportion of PAHs indicative of biomass burning fits the trend of a higher dependence on wood-burning for heating. Our results also show that the transformation of the energy system started to affect the deposition of organic pollutants around 2000. These findings warrant increased efforts to study the implications of increased biomass burning and its effect on the environment and health.

## Supporting Information Available

Core correlation; radiometric dating and chronology; BC quantification method description; PAH identification and quantification—method description; PAH results; grain size analysis and results; BC and SCP concentrations and burial fluxes (PDF)
