# Supplementary material for: Recent Increased Loading of Carbonaceous Pollution from Biomass Burning in the Baltic Sea
Source: ACS Omega. 2022 Sep 23;7(39):35102–8. doi: 10.1021/acsomega.2c04009 (PMC9535721; doi:10.1021/acsomega.2c04009)
Supplement: Supplementary file 1 — ao2c04009_si_001.pdf [file ao2c04009_si_001.pdf]

Supplementary information

Recent increased loading of carbonaceous pollution from biomass burning in the Baltic Sea

Karl Ljung, Petra L. Schoon, Marcus Rudolf, Laurie M. Charrieau, Sha Ni, Helena L. Filipsson

Radiometric dating and chronology

The chronology of the core was based on ^{210}Pb and ^{137}Cs from a parallel core. The two cores were correlated using their TOC patterns (Figure S1) . The dating of the core was previously published and described in Charrieau et al. [1]. Measurements of ^{210}Pb and ^{137}Cs were measured with an ORTEC HPGe (High-Purity Germanium) Gamma Detector at the Department of Geology at Lund University, Sweden. Measured ^{210}Pb values were corrected for self-absorption corrections following Cutshall et al. [2]. The instrument was calibrated against in-house standards and the maximum error was 0.5 years in the measurements. Excess (unsupported) ^{210}Pb was measured down to 23 cm and the age model was calculated based on the Constant Rate of ^{210}Pb Supply (CRS) model[3].

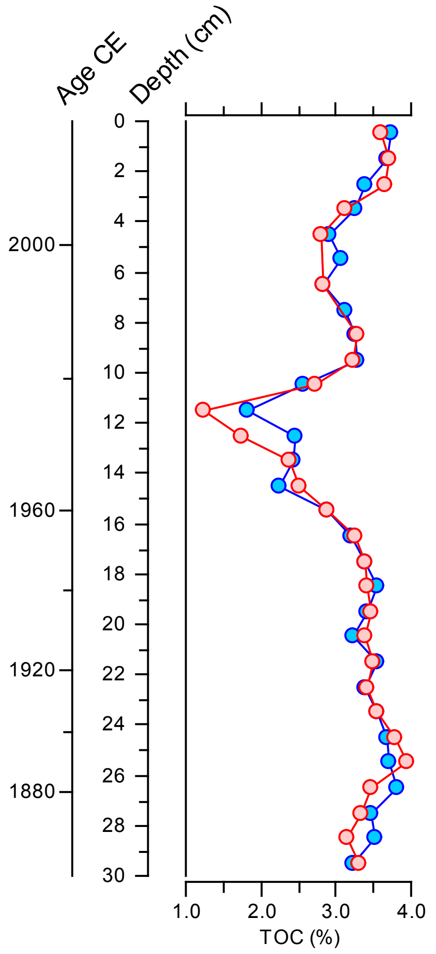

Figure S1: total organic carbon concentrations in the dated core DV-G (blue) and DV-H (red) used in this study. The data is plotted on depth scale with age (CE) as secondary axis.

The unsupported ^{210}Pb in the dated core decreased with depth and allowed direct dating of the core between 2013 and 1913. A ^{137}Cs peak 9 cm depth corresponding to the Chernobyl accident in 1986 was observed (Figure S2). The sedimentation rate decreased from 5.6 mm yr^{-1} at the top of the core to 1 mm yr^{-1} in the bottom. Ages older than 1913 were derived by linear extrapolation based on a sedimentation rate of 1.4 mm yr^{-1}, corresponding to the linear mean sedimentation rate between the years 1913 and 1946.

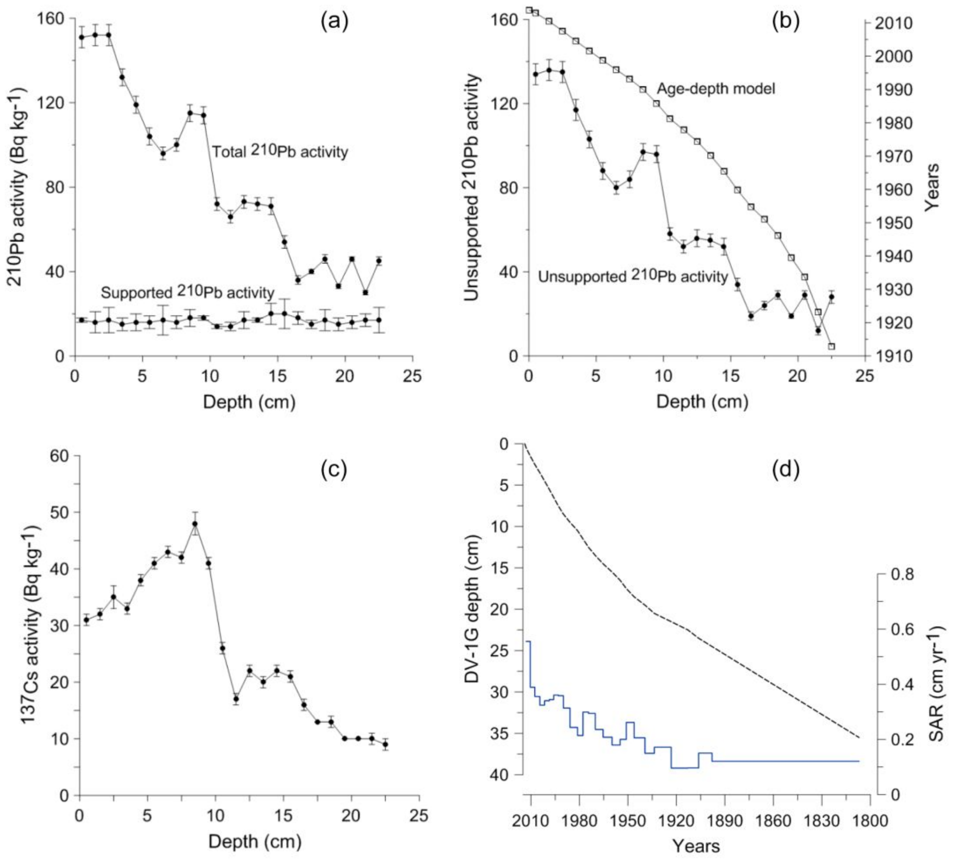

Figure S2. Age–depth model for the sediment sequence from the Öresund (DV-1). (a) Total and supported ^{210}Pb activity. (b) Unsupported ^{210}Pb activity and age model. (c) ^{137}Cs activity. The peak in ^{137}Cs activity corresponds to the Chernobyl accident in 1986. (d) Age–depth model for the sediment sequence based on ^{210}Pb dates and calculated sediment accumulation rates (SARs). Figure from Charrieau et al.[1].

Black carbon quantification

Quantification of BC was done using the chemo thermal oxidation method at 375°C[4–6]. About 20 mg of freeze-dried sediment was weighed into silver capsules. The samples were oxidised in a tube furnace (ENTECH ETF15) with a forced air flow of 250-300 ml/min for 24 h. The temperature in the center of the tube furnace was monitored with an external temperature probe and was within ±2°C throughout the processing. After thermal oxidation, carbonates were removed with acid fumigation

using 12M HCl in a desiccator[6]. Fumigation was used instead of in-capsule acidification to limit potential problems with spitting and material loss during acidification. The remaining carbon of the sediment was analysed with an elemental analyser (COSTECH ECS4010) and defined as black carbon. The elemental analyser performance was checked by running soil reference materials with known carbon content.

The oxidation step was evaluated by analysing BC-free material (wood) and two reference materials with published BC values: diesel soot (SRM2975) and harbor sediment (NIST 1944). The response of the BC-free material showed results indistinguishable from proceeding blanks, thus showing that there was no charring during the oxidation step. The detection limit was estimated to 2 µg C as the mean value of blanks plus three times the standard deviation (n=3). All sample responses were at least one order of magnitude higher than the estimated detection limit. The method precision was estimated as the relative standard deviation of replicated reference material (NIST1944, n=3), and was found to be 15.6 % of reported values. The average values and ranges of SRM2975 and NIST1944 were within the ranges of published results (Table S1).

Tabel S1: Measured and published values of BC concentration in soot (SRM2975) and harbour sediments (NIST1944).

	**BC % (this study)**	**BC % (Reported values)**
SRM2975	68.3±0.5 (n=3)	63±4.1[5] – 68.2±0.9[4] – 68.9±0.79[7] - 64.4±0.8[8]
NIST1944	0.77±0.12 (n=3)	0.66±0.16[4] - 0.8±0.02[4] - 0.96±0.04[8]

Polyaromatic hydrocarbon identification and quantification

Polycyclic hydrocarbon concentration was measured using solvent extraction and gas-chromatography and mass spectrometry. Freeze-dried sediments (~7.5 g) were Soxhlet extracted using a Büchi B-811 Extraction System with a 7.5:1, v/v mixture of dichloromethane (DCM) and methanol (MeOH) and for a duration of 72 cycles. All total lipid extracts (TLEs) were evaporated to near dryness by rotary evaporation and dried over sodium sulphate (Na_2SO_4) to completely remove any traces of water. Elemental sulfur was removed using activated copper. The TLEs were further separated into apolar, aromatic and polar fractions by means of silica gel chromatography using *n*-hexane, 9:1 (v:v) *n*-hexane: DCM, and 1:1 DCM:MeOH solvent mixtures, respectively.

The aromatic fraction containing the PAHs was analysed by gas chromatography (GC) and GC/mass spectrometry (MS). Samples were dissolved in ethyl acetate. GC analyses were performed on an Agilent 7890B instrument equipped with a flame ionisation detector (FID) and with a splitless injector and aA 30m HP-5ms capillary column. The injection was at a temperature of 60 °C which was held for 1 minute. Subsequently, the temperature was ramped to 290 °C at a rate of 3 °C/min and held for 30 min. GC/MS analyses were done on a Shimadzu QP2010 GC/MS system with an HP-5ms capillary column. The gas chromatographic conditions were similar to those described above. Mass spectrometry was performed with an ionisation energy of 70 eV and in full scan mode with a mass range of m/z 50-800 at three scans s^{-1}. Identification of the PAHs was based on comparison with retention times and *m/z* ratio of the mass spectra.

At regular intervals procedural blanks were analysed alongside the sample batches to monitor the quality of the results. Concentrations of PAHs in the blanks were undetectable. The PAHs were quantified using certified reference material purchased from Sigma-Aldrich (Merck) containing 16 PAH compounds (TraceCERT). The calibration of the GC/MS instrument was based on four-point external calibration curves of each individual quantified PAH. The calibration curves for all analysed compounds were linear with correlation coefficients ranging between 0.943 and 0.994. The efficiency

of recovery of the procedure was determined by using a method based on external standards of known amounts of the PAH mixture. PAH recoveries for all analysed calibration concentrations were ranging between 80 % and 149 %. For the calibration concentration that was also used for the analyte targets the recoveries were close to 100% for all analysed PAHs.

Table S2. Concentrations (ng/g dry weight sediment) of polycyclic aromatic hydrocarbons in DV-1. Phen: Phenanthrene, Ant: Anthracene, Fl: Fluoranthene, Pyr: Pyrene, BaA: Benz(a)anthracene, Chr: Chrysene, BbF: Benzo(b)fluoranthene, BkF: Benzo(k)fluoranthene, BaP: Benzo(a)Pyrene, diBahA: Dibenz(a,h)anthracene, BghiP: Benzo(ghi)perylene, IcdP: Indeno(1,2,3-C,D)pyrene. The values of phenantrene and anthracene in sample DV-1 H7 (in italics) are considered as outliers. Samples below detection limit are denoted: n.d.

			3-ring PAHs			4-ring PAHS					5-ring PAHS					6 ring PAHs		
**Sample ID**	**Depth cm bsf**	**Cal age CE**	**Phen**	**Ant**	**∑ 3 ring**	**Fl**	**Pyr**	**BaA**	**Chr**	**∑ 4 ring**	**BbF**	**BkF**	**BaP**	**diBahA**	**∑ 5 ring**	**BghP**	**IcdP**	**∑ 6 ring**
DV-1 H1	0.5	2013	15.5	n.d.	15.5	104.2	87.3	56.6	79.3	327.5	143.1	80.9	84.0	126.7	434.8	n.d.	158.9	4.4
DV-1 H2	1.5	2010	33.5	n.d.	33.5	291.6	231.7	114.8	200.3	838.4	316.9	146.2	131.2	305.1	899.4	n.d.	313.9	8.4
DV-1 H3	2.5	2007	20.5	n.d.	20.5	182.0	141.0	66.1	116.3	505.4	196.4	70.1	84.7	185.3	536.4	n.d.	182.9	5.0
DV-1 H4	3.5	2004	48.6	n.d.	48.6	178.4	146.9	68.3	113.3	507.0	213.0	86.3	101.8	200.8	601.9	n.d.	211.7	6.8
DV-1 H5	4.5	2001	155.5	n.d.	155.5	381.3	284.5	143.0	218.9	1027.6	375.8	158.8	160.5	332.2	1027.3	n.d.	323.2	11.5
DV-1 H6	5.5	1995	22.5	n.d.	22.5	112.8	104.3	51.7	80.7	349.6	155.6	55.4	76.8	137.7	425.5	n.d.	143.4	5.1
DV-1 H7	6.5	1990	*5.3*	*92.4*	*97.7*	273.8	257.7	120.8	177.2	829.6	236.6	107.7	151.7	167.2	663.2	n.d.	216.3	6.6
DV-1 H8	7.5	1986	137.5	11.2	148.7	363.7	346.1	165.0	244.2	1119.0	222.1	130.2	197.9	101.8	651.9	n.d.	228.9	7.1
DV- H10	8.5	1981	56.7	2.7	59.4	184.8	179.0	82.3	126.4	572.5	167.9	80.2	112.1	111.4	471.6	n.d.	178.9	6.5
DV-1 H11	9.5	1978	27.6	2.9	30.6	228.5	241.3	109.0	170.7	749.4	191.2	83.1	143.0	130.3	547.7	n.d.	206.5	16.5
DV-1 H12	10.5	1974	23.6	n.d.	23.6	160.4	128.0	75.0	128.6	492.1	253.9	97.2	73.2	226.7	651.0	n.d.	228.1	13.1
DV-1 H13	11.5	1970	37.6	2.6	40.2	257.6	251.0	125.0	167.2	800.7	132.1	87.6	138.9	71.1	429.7	n.d.	147.2	6.2
DV-1 H14	12.5	1965	32.5	3.7	36.2	207.2	195.8	75.0	92.4	570.4	61.9	33.8	61.4	31.1	188.3	n.d.	64.8	2.6
DV-1 H15	13.5	1960	20.9	1.1	21.9	141.2	153.4	64.6	103.9	463.1	101.0	47.0	101.1	68.1	317.2	n.d.	102.6	3.6
DV-1 H16	14.5	1955	21.0	3.0	24.0	82.9	83.7	46.2	71.5	284.3	103.4	38.7	77.6	86.6	306.4	n.d.	107.1	3.3
DV-1 H17	15.5	1951	6.7	n.d.	6.7	34.7	34.0	16.4	35.3	120.3	40.4	15.5	35.8	30.0	121.8	n.d.	47.7	1.4
DV-1 H18	16.5	1946	n.d.	n.d.	n.d.	27.4	25.7	14.3	36.4	103.8	40.4	16.5	36.8	25.1	118.8	n.d.	42.8	1.3
DV-1 H19	18.5	1934	n.d.	n.d.	n.d.	24.9	19.6	12.5	29.8	86.7	47.7	15.3	n.d.	47.7	110.7	n.d.	56.0	1.6
DV-1 H20	19.5	1923	n.d.	n.d.	0.6	10.7	9.3	7.0	18.5	45.5	20.1	6.9	n.d.	21.1	48.2	n.d.	28.9	0.8
DV-1 H21	20.5	1913	1.6	n.d.	1.6	20.7	14.6	7.1	26.9	69.2	40.0	13.7	20.3	41.5	115.6	5.1	46.4	1.5
DV-1 H22	21.5	1906	n.d.	n.d.	n.d.	13.3	9.1	6.7	25.0	54.2	30.6	9.0	22.6	31.8	94.0	n.d.	39.6	1.1
DV-1 H23	22.5	1898	1.1	n.d.	1.1	19.0	16.0	5.7	7.0	47.6	30.0	4.1	29.1	26.0	89.1	n.d.	38.6	1.0
DV-1 H24	23.5	1890	n.d.	n.d.	n.d.	18.7	13.5	6.6	8.6	47.4	29.6	10.3	n.d.	27.4	67.3	n.d.	38.1	1.0
DV-1 H25	24.5	1881	n.d.	n.d.	n.d.	15.8	11.4	7.3	9.2	43.7	22.3	8.0	n.d.	18.4	48.7	n.d.	29.0	0.8
DV-1 H26	25.5	1873	n.d.	n.d.	n.d.	9.4	7.3	2.7	11.3	30.8	28.3	6.2	11.6	24.4	70.5	n.d.	31.4	0.9
DV-1 H27	26.5	1865	n.d.	n.d.	n.d.	7.1	5.6	4.0	7.0	23.7	16.3	5.3	n.d.	15.9	37.5	n.d.	21.8	0.7
DV-1 H28	27.8	1857	n.d.	n.d.	n.d.	7.4	6.1	5.4	15.2	34.1	16.9	6.2	n.d.	14.4	37.4	n.d.	24.1	0.7

Grain size

Details on the grain size analysis method and data can be found in Charrieau (2019). Briefly, the samples were treated with H2O2, HCl, and NaOH to remove organic matter, carbonates and biogenic silica. The sand fraction (> 63 µm) was separated by sieving and the fine fractions < 63 µm was analyzed by laser diffraction using a Sedigraph III Particle Size Analyzer.

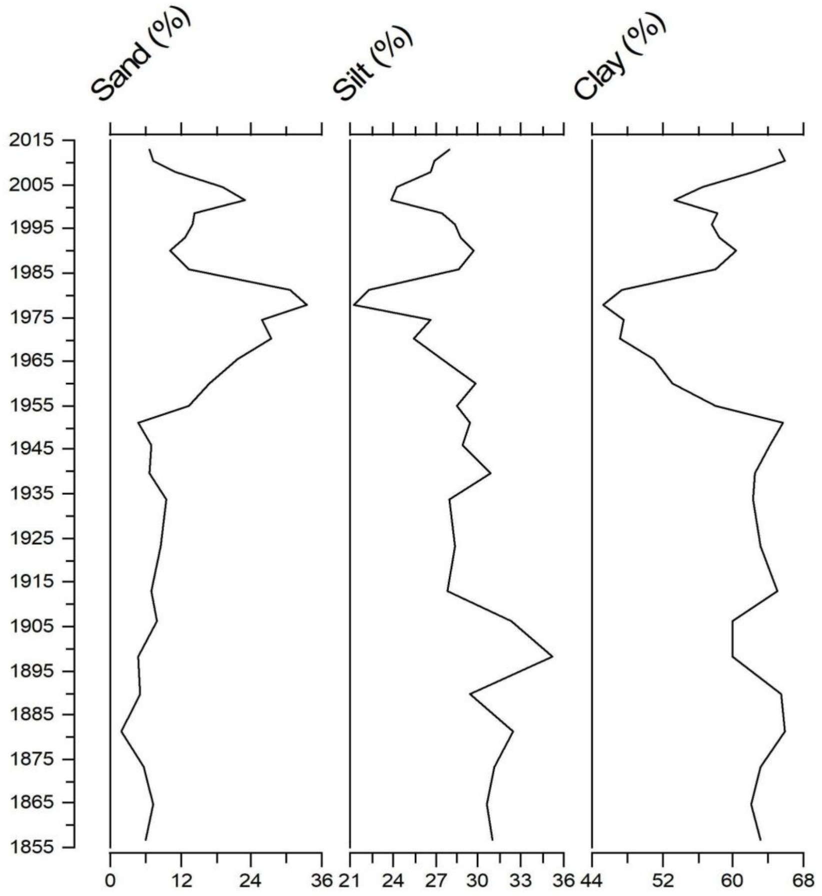

Figure S3: Grain size distribution in DV-1 core.

Table S3: black carbon (BC) and spheriodial carbonaceous particle (SCP) concentrations and burial fluxes. Samples with no detected SCPs are denoted n.d.

Depth	Age CE	BC (%)	BC burial flux µg/cm^2/yr	SCP/gdw	SCP burial flux particles/cm^2/yr
**0.5**	2013	0.20	419	24276	5083
**1.5**	2010	0.19	344	42989	7622
**2.5**	2008	0.19	352	35092	6590
**3.5**	2005	0.18	497	15489	4243
**4.5**	2002	0.16	383	15004	3635
**6.5**	1996	0.18	463	18279	4627
**8.5**	1990	0.19	313	20105	3388
**9.5**	1986	0.20	310	17713	2758
**10.5**	1981	0.16	321	19265	3811
**11.5**	1978	0.07	186	17684	5056
**12.5**	1974	0.11	214	27610	5631
**13.5**	1970	0.16	268	13073	2225
**14.5**	1965	0.16	179	13260	1498
**15.5**	1960	missing	missing	10443	1599
**16.5**	1955	0.23	374	2717	441
**17.5**	1951	0.24	317	2747	370
**18.5**	1946	0.28	264	1265	121
**19.5**	1939	0.23	239	1841	187
**20.5**	1934	0.27	172	612	40
**21.5**	1923	0.25	166	945	63
**22.5**	1913	0.24	122	475	25
**23.5**	1906	0.27	167	n.d.	n.d.
**24.5**	1898	0.27	189	372	27
**25.5**	1890	0.28	176	n.d.	n.d.
**26.5**	1881	0.28	230	158	13
**27.5**	1873	0.23	180	918	70
**28.5**	1865	0.24	167	n.d.	n.d.
**29.5**	1857	0.24	216	n.d.	n.d.

References

(1) Charrieau. L. M.; Ljung. K.; Schenk. F.; Daewel. U.; Kritzberg. E.; Filipsson. H. L. Rapid Environmental Responses to Climate-Induced Hydrographic Changes in the Baltic Sea Entrance. *Biogeosciences* **2019**. *16* (19). 3835–3852. https://doi.org/10.5194/bg-16-3835-2019.

(2) Cutshall. N. H.; Larsen. I. L.; Olsen. C. R. Direct Analysis of 210Pb in Sediment Samples: Self-Absorption Corrections. *Nuclear Instruments and Methods in Physics Research* **1983**. *206* (1). 309–312. https://doi.org/10.1016/0167-5087(83)91273-5.

(3) Appleby. P. G. Chronostratigraphic Techniques in Recent Sediments. In *Tracking Environmental Change Using Lake Sediments: Basin Analysis. Coring. and Chronological Techniques*; Last. W.

M.. Smol. J. P.. Eds.; Developments in Paleoenvironmental Research; Springer Netherlands: Dordrecht. 2001; pp 171–203. https://doi.org/10.1007/0-306-47669-X_9.

(4) Gustafsson. Ö.; Bucheli. T. D.; Kukulska. Z.; Andersson. M.; Largeau. C.; Rouzaud. J.-N.; Reddy. C. M.; Eglinton. T. I. Evaluation of a Protocol for the Quantification of Black Carbon in Sediments. *Global Biogeochemical Cycles* **2001**. *15* (4). 881–890.

(5) Nguyen. T. H.; Brown. R. A.; Ball. W. P. An Evaluation of Thermal Resistance as a Measure of Black Carbon Content in Diesel Soot. Wood Char. and Sediment. *Organic Geochemistry* **2004**. *35* (3). 217–234. https://doi.org/10.1016/j.orggeochem.2003.09.005.

(6) Agarwal. T.; Bucheli. T. D. Adaptation. Validation and Application of the Chemo-Thermal Oxidation Method to Quantify Black Carbon in Soils. *Environmental Pollution* **2011**. *159* (2). 532–538. https://doi.org/10.1016/j.envpol.2010.10.012.

(7) Ruppel. M. M.; Gustafsson. Ö.; Rose. N. L.; Pesonen. A.; Yang. H.; Weckström. J.; Palonen. V.; Oinonen. M. J.; Korhola. A. Spatial and Temporal Patterns in Black Carbon Deposition to Dated Fennoscandian Arctic Lake Sediments from 1830 to 2010. *Environ. Sci. Technol.* **2015**. *49* (24). 13954–13963. https://doi.org/10.1021/acs.est.5b01779.

(8) Louchouarn. P.; Chillrud. S. N.; Houel. S.; Yan. B.; Chaky. D.; Rumpel. C.; Largeau. C.; Bardoux. G.; Walsh. D.; Bopp. R. F. Elemental and Molecular Evidence of Soot- and Char-Derived Black Carbon Inputs to New York City's Atmosphere during the 20th Century. *Environ. Sci. Technol.* **2007**. *41* (1). 82–87. https://doi.org/10.1021/es061304+.
